# Differential regulation of the two transcriptional activation domains of the coiled-coil coactivator CoCoA by sumoylation

**DOI:** 10.1186/1471-2199-9-12

**Published:** 2008-01-25

**Authors:** Catherine K Yang, Jeong Hoon Kim, David K Ann, Michael R Stallcup

**Affiliations:** 1 Department of Biochemistry and Molecular Biology and the Norris Comprehensive Cancer Center, University of Southern California, Los Angeles, California 90089-9176, USA; 2 Department of Clinical and Molecular Pharmacology, Beckman Research Institute, City of Hope, Duarte California 91010, USA

## Abstract

**Background:**

The coiled-coil coactivator (CoCoA) enhances transcriptional activity of nuclear receptors, the xenobiotic aryl hydrocarbon receptor, and the lymphocyte enhancer factors (LEF) in the Wnt/β-catenin signaling pathway. CoCoA is comprised of a large central coiled coil domain flanked by N-terminal and C-terminal activation domains (AD). The N-terminal AD of CoCoA is required for coactivator function with LEF and β-catenin, while the C-terminal AD of CoCoA is required for coactivator function with nuclear receptors. We explored the role of sumoylation in regulating the activities of the two ADs and the coactivator function of CoCoA.

**Results:**

The N-terminus of CoCoA is covalently modified by SUMO1 at Lys-29; both PIAS1 and ARIP3 function as E3 ligases. Fusion of SUMO1 to the N-terminus (mimicking sumoylation) reduced coactivator function of CoCoA with LEF1 and the activity of the N-terminal AD. The N- and C-termini of CoCoA can bind to each other, and C-terminal transactivation activity is attenuated in the presence of the N-terminus, indicating that the N-C interaction regulates the activity of the C-terminal AD. Fusion of SUMO1 to the N-terminal fragment of CoCoA reduced the N-C interaction and inhibition of C-terminal AD activity by the N-terminal fragment.

**Conclusion:**

Sumoylation of CoCoA differentially regulates the coactivator activity of CoCoA with nuclear receptors versus LEF1, by attenuating the N-terminal AD activity and enhancing the activity of the C-terminal AD.

## Background

Post-translational modification of proteins is a widely used mechanism for regulation of a variety of cellular processes. Modification by the ubiquitin homologue, the small ubiquitin-like modifier 1 (SUMO1, also known as sentrin, GMP1, UBL1, and PIC1) [1], has been identified as an essential mechanism for the dynamic regulation of gene expression, cell cycle progression, DNA repair, and cellular protein trafficking [2,3]. There are three mammalian SUMOs, SUMO1, SUMO2 and SUMO3. SUMO1 consists of 101 amino acids, and shares approximately 50% sequence identity with SUMO2 and SUMO3, and with the yeast Smt 3 protein [4]. Ligation of SUMO1 to specific lysine residues of target proteins is a multi-step process which resembles ubiquitin conjugation. SUMO1 is initially made as an inactive precursor which matures by an ATP-dependent carboxy-terminal proteolytic cleavage event, catalyzed by the E1 activation enzyme complex containing the UBA2 and AOS1 proteins. Activated SUMO1 is subsequently attached covalently to the E2 conjugating enzyme Ubc9, and finally conjugated to the ε-amino group of specific lysine residues of target proteins by an E3 ligase. While there is only one known E2 conjugating enzyme, many E3 ligases for sumoylation have been identified. These include the polycomb protein Pc2 [5], RanBP2 [6], and members of the PIAS family, which includes PIAS1, PIAS3, PIASy, PIASxα/ARIP3, PIASxβ/Miz3 and hZimp10 [7,8]. In addition to their E3 ligase acitivity, PIAS1 has been shown to activate transcription factors such as p53 by a mechanism independent of ligase activity [9].

The physiological effects of protein sumoylation are extremely varied. Sumoylation is involved in cytokine signaling by attenuating the activity of the activated forms of STAT1, which is responsible for inducing the expression of specific genes in response to cytokines [10,11]. Sumoylation of the PIASy protein, which subsequently promotes sumoylation of lymphocyte enhancer factor 1 (LEF1) in the developmentally important Wnt/β-catenin signaling pathway, results in sequestration of LEF1 to the PML nuclear bodies and inhibition of LEF1-mediated transcription [12]. Sumoylation of a number of nuclear receptors has also been demonstrated and generally results in inhibition of these transcriptional activators [13-15].

In this study, we focus on the functional significance of sumoylation of the coiled-coil coactivator (CoCoA). Although CoCoA was originally discovered as a coactivator for nuclear receptors [16] and the aryl hydrocarbon receptor [17], it is also involved in the transcriptional activation of target genes by LEF/T cell factor (TCF) transcription factors, some of which are controlled by the Wnt/β-catenin pathway [18]. Wnt ligands lead to stabilization and increased cellular levels of β-catenin, which enters the nucleus and binds to and serves as a coactivator for LEF/TCF transcription factors [19]. LEF/TCF transcription factors bind to specific DNA sequences which serve as enhancer elements controlling the expression of specific genes. DNA-bound LEF/TCF recruits the coactivator β-catenin, which functions as a scaffold for binding of other coactivators, including CBP/p300, p160 coactivators, protein arginine methyltransferase CARM1, and CoCoA [20].

CoCoA consists of a large central coiled-coil domain, flanked by N-terminal and C-terminal activation domains (ADs). Both the N-terminal and C-terminal ADs of CoCoA interact with the C-terminal region of β-catenin. The N-terminal AD of CoCoA is necessary and sufficient to bind to and cooperate with β-catenin as a coactivator for LEF/TCF proteins in transient transfection assays; the C-terminal AD of CoCoA is not required [20]. In contrast, when CoCoA functions as a coactivator for nuclear receptors, the functional domain requirements are completely different. Hormone activated nuclear receptors bind specific DNA enhancer elements and recruit p160 coactivators, which serve as scaffolds for binding of a variety of coactivators, including CBP/p300, CARM1, and CoCoA [21]. The coiled-coil domain of CoCoA binds to the N-terminal region of the p160 coactivator; the C-terminal AD of CoCoA, but not the N-terminal AD, is required for the coactivator function of CoCoA with nuclear receptors [16,18]. The mechanisms of action of each AD of CoCoA are unique and complex, but one common element of their activity involves the binding of CBP/p300 to a specific amino acid motif that occurs in both ADs [20,22]. Because CoCoA uses different activation domains in its coactivator functions with nuclear receptors and LEF1, differential regulation of the relative activities of the N-terminal and C-terminal ADs of CoCoA could differentially modulate the ability of CoCoA to enhance the function of LEF/TCF transcription factors versus nuclear receptors.

Here we report that CoCoA is SUMO-modified *in vitro *and *in vivo*. Sumoylation of CoCoA is stimulated by the SUMO E3 ligases PIAS1 and ARIP3. Mutation of the major sumoylation site of CoCoA or fusion of SUMO1 to the N-terminus of CoCoA (mimicking the natural sumoylation process) altered the activity of CoCoA N-terminal AD and the coactivator function of CoCoA. Interestingly, while wild type CoCoA N-terminal AD can bind to and repress the activity of the CoCoA C-terminal AD, the SUMO1 fusion to CoCoA N-terminus attenuated the interaction. Taken together, our data suggest that sumoylation of CoCoA regulates the differential use of the ADs by attenuating the N-terminal AD activity and by enhancing the activity of the CoCoA C-terminal AD. Thus, sumoylation may favor CoCoA coactivator function with nuclear receptors versus LEF1/β-catenin.

## Results

### Sumoylation of CoCoA *in vivo *and *in vitro*

**Figure 1:**
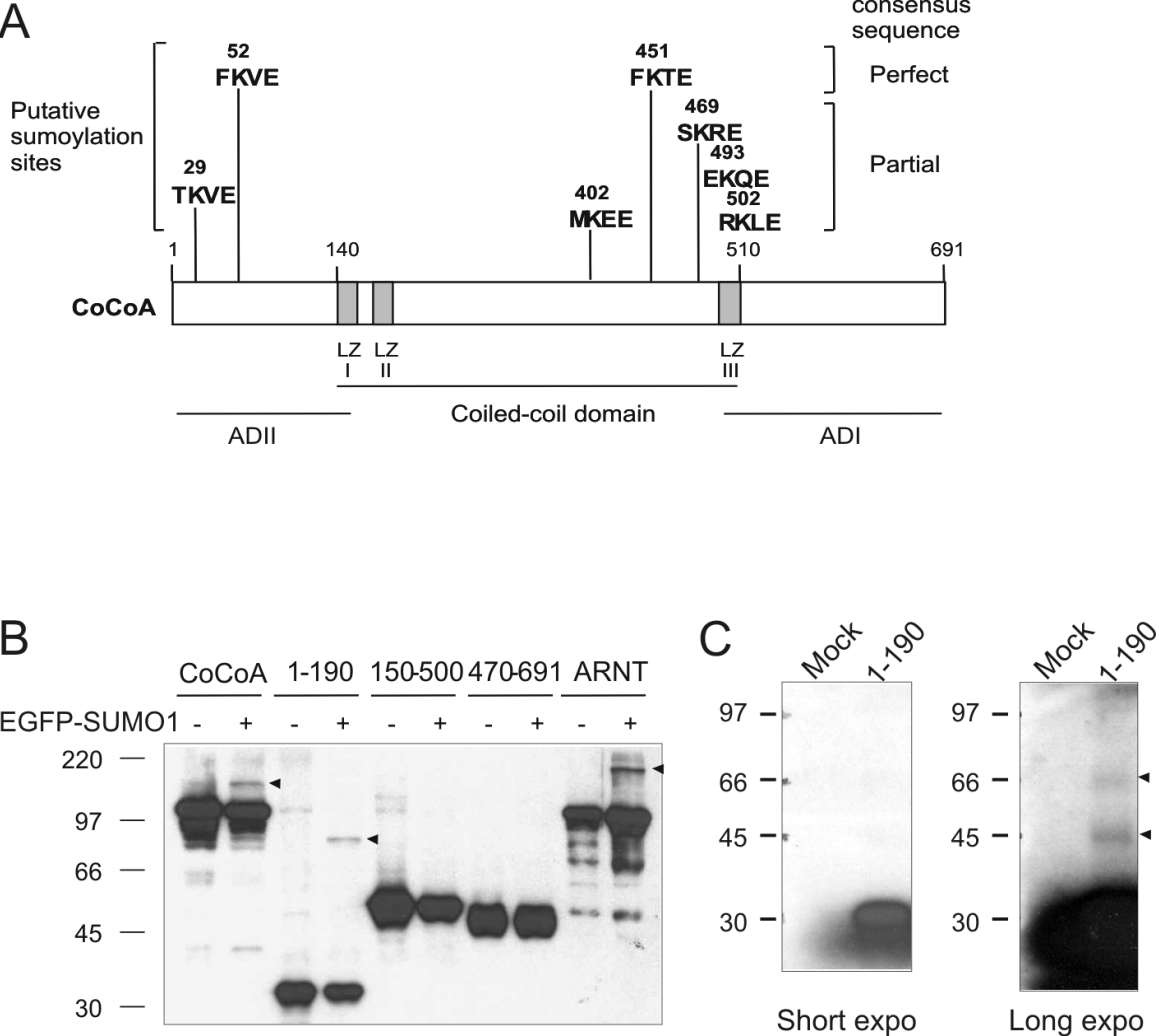
**Sumoylation of CoCoA**. (A) Schematic diagram of predicted sumoylation sites of CoCoA. AD, activation domain; LZ, leucine zipper-like sequence. (B) *In vivo *sumoylation of CoCoA. COS-7 cells were transfected with pSG5.HA.ARNT, pSG5.HA.CoCoA full-length, or pSG5.HA.CoCoA deletion mutants (800 ng); an EGFP-SUMO1 expression vector (200 ng) was co-transfected, where indicated. Cell extracts were collected 48 h post-transfection and analyzed by immunoblot with anti-HA antibody. Arrowheads indicate apparent sumoylation products. (C) *In vitro *sumoylation of CoCoA. *In vitro *translated ^35^S-labeled CoCoA(1–190) was incubated with purified sumoylation E1 (Aos1/Uba2 heterodimer) and E2 (Ubc9) enzymes in the presence of ATP/Mg^2+ ^and SUMO1. In the mock reaction, the CoCoA was omitted. Products were analyzed by SDS-PAGE and autoradiography.

Many transcription factors and cofactors have been identified as sumoylation targets. We therefore examined the possibility of post-transcriptional modification of CoCoA by SUMO1 conjugation. Inspection of the amino acid sequence of CoCoA for the sumoylation concensus sequence ψKxD/E (where ψ is any hydrophobic amino acid and x is any amino acid) revealed several putative sumoylation sites at the N-terminus and at the C-terminal end of the coiled-coil domain (Fig. 1A). We next tested whether CoCoA can be covalently modified by SUMO1. COS-7 cells were transiently transfected with HA-tagged CoCoA and SUMO1 fused to enhanced green fluorescent protein (EGFP). Immunoblot analysis of the cell extracts with anti-HA antibody revealed a major ~100 kDa band corresponding to the unmodified full-length CoCoA and a less intense, more slowly migrating band when full-length CoCoA was co-expressed with EGFP-SUMO1 (Fig. 1B). This slow migrating band was not observed when CoCoA was expressed without EGFP-SUMO1. A slow migrating band was also observed when the N-terminal fragment of CoCoA (amino acids 1–190) was expressed with EGFP-SUMO1, but not in the absence of EGFP-SUMO1. In contrast, no additional slow-moving band was observed when the coiled-coil region (amino acids 150–500) or the C-terminus (amino acids 470–691) of CoCoA was co-expressed with EGFP-SUMO1. The ARNT protein, which is a confirmed sumoylation target [23], also generated a slow migrating band when co-expressed with EGFP-SUMO1 and thus served as a positive control for this *in vivo *sumoylation assay (Fig. 1B). When the ^35^S-labeled N-terminal fragment of CoCoA (amino acids 1–190) was produced by in vitro translation and incubated in a cell free sumoylation reaction, slow-migrating bands were observed, as expected for sumoylated CoCoA N-terminal fragment (Fig. 1C). These results suggest that CoCoA is modified by SUMO1 *in vitro *and *in vivo *within the N-terminal region. It is clear from the data that only a small fraction of CoCoA is sumoylated at any given time.

### Enhancement of CoCoA sumoylation by PIAS1 and ARIP3

**Figure 2:**
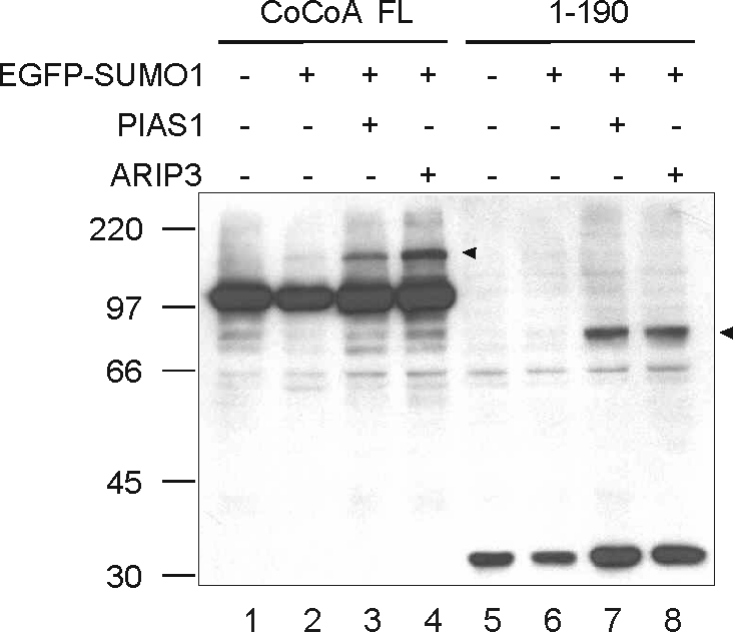
**PIAS1 and ARIP3 act as SUMO E3 ligases for CoCoA**. COS-7 cells were co-transfected with plasmids expressing HA-CoCoA or HA-CoCoA(1–190) (800 ng), EGFP-SUMO1 (200 ng), and PIAS1 or ARIP3 (100 ng) as indicated. Cell extracts were collected 48 h post-transfection and analyzed by immunoblot with anti-HA antibody. Arrowheads indicate the SUMO-conjugated CoCoA or CoCoA(1–190).

A number of proteins have been identified as SUMO E3 ligases; when over-expressed, these proteins can enhance the degree of substrate sumoylation in cells. We tested two known E3 ligases, PIAS1 and ARIP3/PIASxα, which are known to enhance sumoylation of other transcription coactivators, including the p160 coactivator GRIP1 [24]. As shown above (Fig. 1B), co-expression of full-length CoCoA or the N-terminal fragment of CoCoA with EGFP-SUMO1 produced a faint, slow-migrating species (Fig. 2, lanes 2 and 6). Over-expression of PIAS1 or ARIP3 substantially enhanced the amount of the higher molecular weight CoCoA species (lanes 3, 4, 7, and 8), suggesting that PIAS1 and ARIP3 can act as E3 ligases for sumoylation of CoCoA. Furthermore, co-immunoprecipitation assays showed that CoCoA interacts with PIAS1 *in vivo *(data not shown). The enhanced intensity of the high molecular weight bands in the presence of known E3 ligases also confirms that these higher molecular weight bands are indeed SUMO-conjugated forms of CoCoA.

### Identification of the sumoylation site in CoCoA

**Figure 3:**
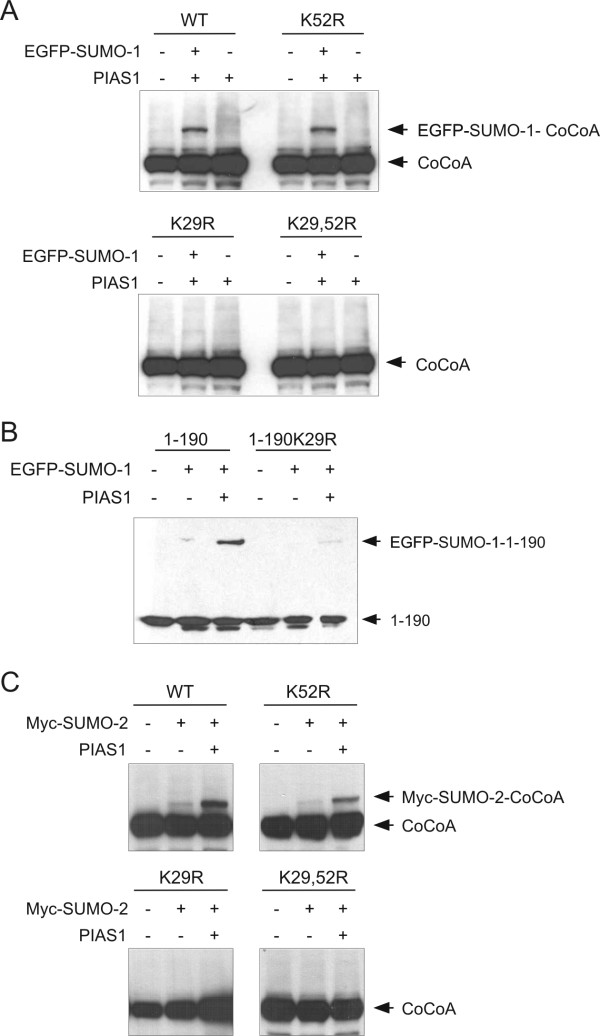
**Identification of SUMO1 conjugation site in CoCoA**. (A) COS-7 cells were co-transfected with plasmids expressing EGFP-SUMO1 (200 ng), PIAS1 (100 ng), and full-length HA-CoCoA wild type or mutants (800 ng) as indicated. (B) 293T cells were transfected with plasmids expressing EGFP-SUMO1 (200 ng), PIAS1 (100 ng), and HA-CoCoA(1–190) wild type or K29R mutant (800 ng) as indicated. Cell extracts were collected 48 h after transfection and analyzed by immunoblot with anti-HA antibody. (C) COS-7 cells were co-transfected with plasmids expressing Myc-SUMO2 (200 ng), PIAS1 (100 ng), and full-length HA-CoCoA wild type or mutants (800 ng) as indicated. Cell extracts were collected 48 h after transfection and analyzed by immunoblot with anti-HA antibody.

Sumoylation occurs at lysine residues within a specific sequence context. CoCoA contains a number of putative sumoylation sites (Fig. 1A). However, only two of the putative sites are located in the N-terminal region (amino acids 1–190) which was shown above (Figs. 1 and 2) to undergo sumoylation in vivo. Of these two predicted sumoylation sites, the sequence around Lys-52 (FKVE) perfectly matches the accepted consensus sumoylation sequence, while the sequence around Lys-29 (TKVE) partially matches the consensus sequence. We tested whether mutation of either Lys-29 or Lys-52 to arginine (K29R and K52R) would affect the sumoylation status of CoCoA. In immunoblot analyses of extracts from COS-7 cells expressing wild type or mutant CoCoA (full length), the sumoylation of the K29R mutant of CoCoA was dramatically reduced, compared with wild type CoCoA; however, in multiple experiments a very weak band was still observed at the correct position for sumoylated CoCoA (Fig. 3A). However, mutation of K52R, which lies in a perfect sumolyation consensus sequence, caused little or no reduction in the sumoylation of CoCoA. Double mutation at both K29 and K52 further reduced or eliminated the sumoylated CoCoA species. In the N-terminal fragment of CoCoA (amino acids 1–190) the K29R mutation caused a similar reduction in sumoylation (Fig. 3B). These results indicate that K29 is the major site of sumoylation in CoCoA, while K52 may be a minor sumoylation site. We next tested whether CoCoA can be modified by SUMO2. As shown in Fig. 3C, CoCoA was efficiently conjugated to SUMO2. Mutation at K29 also abolished CoCoA sumoylation by SUMO2. These results confirmed that K29 is the major sumoylation site of CoCoA.

### Effect of sumoylation on the function of CoCoA N-terminal AD

**Figure 4:**
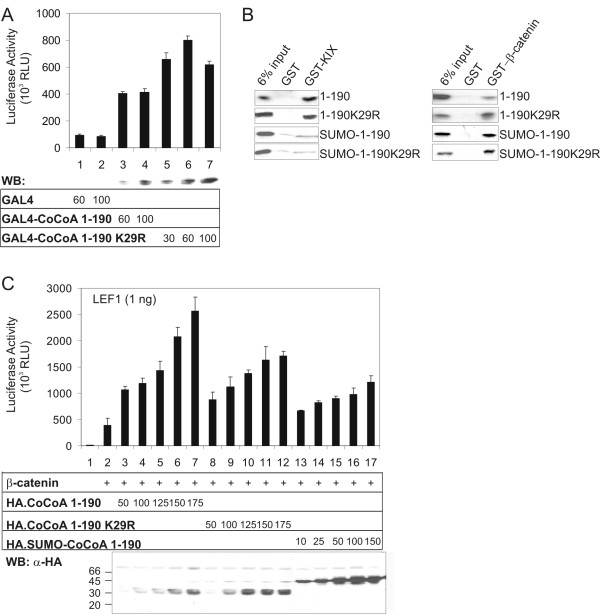
**Effect of sumoylation on the activity of CoCoA N-terminal AD**. (A) 293T cells were transfected in 24-well plates with GK1-Luc reporter plasmid (150 ng) and plasmids encoding Gal4 DBD or Gal4 DBD-CoCoA fusion proteins (30, 60 or 100 ng) as indicated. Cell extracts were assayed for luciferase activity or analyzed by immunoblot with anti-Gal4 antibody 48 h after transfection. Luciferase activity results shown are from a single experiment which is representative of four independent experiments. (B) *In vitro*-translated HA-tagged CoCoA(1–190) wild type or mutant was incubated with GST, GST-p300 KIX fragment (left panel) or GST-β-catenin (right panel) fusion proteins bound to glutathione-Sepharose. Bound proteins were eluted and analyzed by immunoblot with anti-HA antibody (left panel) or autoradiography (right panel). (C) 293T cells were transfected in 24-well plates with pGL3OT reporter plasmid (100 ng), pSG5.HA-LEF1 (1 ng), pSG5.HA-β-catenin (1 ng), and a pSG5.HA vector encoding CoCoA(1–190), CoCoA(1–190, K29R), or SUMO1-fused CoCoA(1–190) (10–175 ng) as indicated. Cell extracts were assayed for luciferase activity or analyzed by immunoblot with anti-HA antibody 48 h after transfection. Luciferase activity results shown are from a single experiment which is representative of two independent experiments. RLU, Relative light units.

Sumoylation has been found to regulate protein function in a variety of ways, including effects on cellular localization, protein-protein interactions, and transcriptional activation activity. Mammalian two-hybrid and co-immunoprecipitation assays failed to detect any difference in the ability of wild-type CoCoA and the K29R mutant of CoCoA to homo-dimerize or interact with GRIP1 (data not shown). In addition, no difference in the cellular distribution of wild-type and K29R mutant CoCoA was observed by confocal microscopy (data not shown). However, when the wild type or mutant CoCoA N-terminal AD fragment was fused to GAL4 DNA binding domain (DBD), the K29R mutant displayed a moderately increased autonomous activation function compared to the wild type AD fragment (Fig. 4A, assays 3–7). Similar quantities of the wild type and mutant expression vectors produced different amounts of CoCoA protein. When the activities of similar amounts of wild type and mutant proteins were compared, the mutant protein was moderately more active (e.g. assay 4 versus 5 and 6).

Previously we have shown that CoCoA functions as a secondary coactivator for LEF1. LEF1, bound to its specific enhancer element, recruits β-catenin, which serves as a molecular scaffold for recruitment of various secondary coactivators, such as CBP/p300, CARM1, and CoCoA [18]. The N-terminal AD of CoCoA is important for this coactivator function with LEF1/β-catenin for two reasons: it contains a binding site for β-catenin and it also contains the AD function which contributes to the overall process of transcriptional activation [20]. Surprisingly, deletion of the C-terminal AD did not compromise the ability of CoCoA to cooperate with β-catenin as a coactivator for LEF1, and even the N-terminal fragment (amino acids 1–190) alone cooperated with β-catenin as a coactivator for LEF1. Although we do not know the complete mechanism by which the CoCoA N-terminal domain helps to promote formation of an active RNA polymerase II complex on the promoter, we have shown that one important element of the N-terminal AD is a binding motif for the KIX domain of p300 located at amino acids 17–24 of CoCoA. Point mutations in this motif of CoCoA disrupt p300 binding, transactivation activity of the AD, and coactivator function of CoCoA with LEF1 and β-catenin [20].

Since this p300 KIX binding domain is physically close to the major sumoylation site (K29), we tested the effect of sumoylation on CoCoA interaction with p300 KIX. Because there is no obvious way to achieve a high level of sumoylation at K29 of CoCoA, we instead mimicked the sumoylation at K29 by fusing SUMO1 to the N-terminal end of the N-terminal AD fragment of CoCoA (amid acids 1–190). To prevent removal of this fused SUMO by SUMO-specific proteases, the fused SUMO1 lacked the two tandem glycine residues found at the C-terminus of naturally processed SUMO1. A GST-pull down assay showed that SUMO1 fusion to the N-terminal fragment of CoCoA (amino acids 1–190) substantially reduced the interaction of the N-terminal AD with GST-p300 KIX (Fig. 4B). In contrast, the fusion of SUMO1 to CoCoA did not reduce the interaction between the N-terminal fragment of CoCoA and β-catenin. The K29R mutation had little or no effect on the binding of CoCoA N-terminal AD to either GST-KIX or GST-β-catenin.

**Figure 5:**
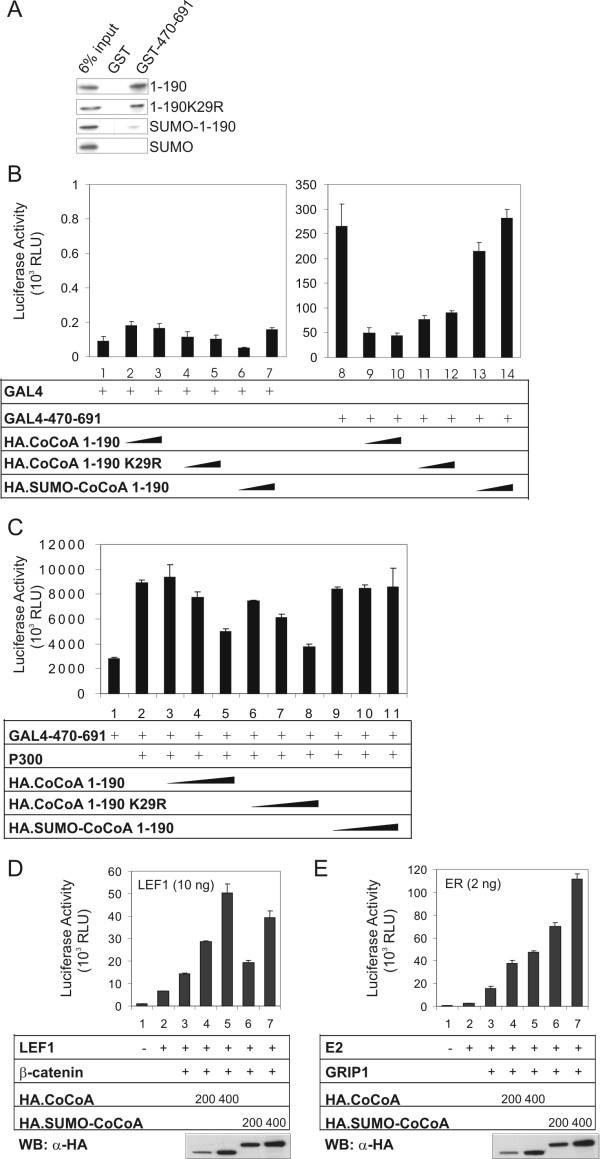
**Effect of sumoylation on the activity of CoCoA C-terminal AD**. (A) *In vitro*-translated CoCoA(1–190) wild type, CoCoA(1–190, K29R), SUMO1-CoCoA(1–190) fusion protein, or SUMO1 were incubated with GST or GST-CoCoA(470–691) fusion protein bound to glutathione-Sepharose. Bound proteins were eluted and analyzed by autoradiography. (B) CV1 cells were transfected in 24-well plates with GK1-Luc reporter plasmid (150 ng) and plasmids encoding Gal4 DBD or Gal4 DBD-CoCoA(470–691) (50 ng), and HA-CoCoA(1–190) wild type or K29R mutant or SUMO1-CoCoA(1–190) (100 or 150 ng), as indicated. Cell extracts were assayed for luciferase activity 48 h after transfection. Luciferase activity results shown are from a single experiment which is representative of four independent experiments. (C) 293T cells were transfected in 24-well plates with GK1-Luc reporter plasmid (150 ng) and plasmids encoding Gal4 DBD-CoCoA(470–691) (50 ng), p300 (25 ng), and pSG5.HA-CoCoA(1–190) wild type or K29R mutant or SUMO1-CoCoA(1–190) (50, 100 or 150 ng) as indicated. Cell extracts were assayed for luciferase activity 48 h after transfection. Results shown are representative of two independent experiments. (D) CV-1 cells were transfected in 12-well plates with pGL3OT reporter plasmid (200 ng), pSG5.HA-LEF1 (10 ng), pSG5.HA-β-catenin (50 ng), and a pSG5.HA vector encoding CoCoA or SUMO1-fused CoCoA, as indicated. Results shown are representative of three independent experiments. (E) CV-1 cells were transfected in 12-well plates with MMTV(ERE)-LUC reporter plasmid (200 ng), pHE0 (2 ng), pSG5.HA-GRIP1 (50 ng), and a pSG5.HA vector encoding CoCoA or SUMO1-fused CoCoA, as indicated. Results shown are representative of four independent experiments. For immunoblots CV-1 cells do not produce enough protein, so COS-7 cells were transfected with 200 or 400 ng of CoCoA expression plasmids. Cell extracts were subjected to immunoblot analysis with anti-HA antibody.

As we showed previously [20], CoCoA(1–190) can function as a coactivator for LEF1 in the presence of β-catenin (Fig. 4C, assays 1–7). This secondary coactivator activity of CoCoA(1–190) in LEF1 mediated transcriptional activation was reduced moderately by the K29R mutation (assays 8–12). The fact that the K29R mutation moderately enhanced the activity of the Gal4 DBD-CoCoA(1–190) fusion protein (Fig. 4A) but moderately decreased the activity of CoCoA(1–190) as a coactivator for LEF1 and β-catenin (Fig. 4C) suggests that Lys-29 has a role in the coactivator function of CoCoA that is not important for the activity of the Gal4-CoCoA fusion protein. The coactivator activity of CoCoA(1–190) was strongly reduced by fusion of SUMO1 to CoCoA (1–190) (assays 13–17), even though the SUMO1 fusion protein was expressed at much higher levels than the unmodified CoCoA(1–190) fragment. (Compare activities in assay 7 versus assays 13–14, where equivalent amounts of CoCoA(1–190) and SUMO-CoCoA(1–190) were expressed). Similar results were obtained with full length CoCoA containing the K29R mutation or an N-terminal fusion to SUMO1 (Fig. 5D and data not shown). These results suggest that even though SUMO addition may increase CoCoA protein synthesis or stability, sumoylation of CoCoA negatively regulates CoCoA N-terminal transcriptional activity.

### Effect of sumoylation on the function of CoCoA C-terminal AD

When hormone-activated nuclear receptors bind to enhancer elements associated with the genes they regulate, the nuclear receptors recruit p160 coactivators, which serve as a scaffold for binding of other coactivators, such as CBP/p300, CARM1, and CoCoA [16]. Previously we have shown that the C-terminal AD of CoCoA contains a strong transactivation activity which is absolutely necessary for its cooperation with the p160 proteins as a coactivator for nuclear receptors. Although this C-terminal fragment of CoCoA exhibits a very powerful activity when fused to Gal4 DBD, full length CoCoA has little or no transactivation activity when fused to Gal4 DBD, suggesting that the N-terminal region somehow inhibits the activity of the C-terminal AD [16]. Here we show that the N-terminal and C-terminal domains of CoCoA can physically interact (Fig. 5A), which may explain the mechanism of inhibition of the C-terminal AD by the N-terminal region. We therefore tested whether sumoylation of the N-terminal region of CoCoA may affect the physical interaction between the termini. Fusion of SUMO1 to the N-terminus of CoCoA(1–190) dramatically decreased its binding to the C-terminal AD. In contrast, the K29R mutant of CoCoA(1–190) bound to the C-terminal AD at wild type levels (Fig. 5A). Thus, sumoylation interferes with the interaction between the N- and C-terminal regions of CoCoA.

We further tested the effect of CoCoA N-C interaction on the C-terminal transcriptional activation activity of CoCoA. When fused to GAL4-DBD, the strong transactivation activity of CoCoA C-terminal AD (amino acids 470–691) (Fig 5B, assay 8) was strongly repressed by co-expression of the wild type or K29R mutant of the CoCoA N-terminal fragment (amino acids 1–190) (assays 9–12). In contrast, the SUMO-CoCoA(1–190) fusion protein had little or no effect on activity of the C-terminal AD (assays 13–14), consistent with its reduced ability to bind to the C-terminal AD (Fig. 5A). These results suggest that the strong repression by the CoCoA N-terminal fragment is due to direct N-C interaction, which may block the ability of the C-terminal AD to interact with and stimulate the activity of other components of the transcription machinery. Sumoylation appears to relieve the repression of the C-terminal AD by the N-terminal domain.

Our previous studies have shown that p300 can directly interact with both the N- and C-termini of CoCoA, and this binding is important for the activities of both ADs [20,22]. Here we show that while p300 enhanced the transcriptional activity of CoCoA C-terminal AD (470–691), co-expression of the wild type or K29R mutant of CoCoA(1–190) reduced the enhancement of C-terminal AD activity by p300 (Fig. 5C), possibly by binding to the C-terminal AD and blocking the binding of p300. In contrast, the SUMO-fusion of the CoCoA N-terminus did not block the p300 effect. The differential activities of CoCoA(1–190) and SUMO1-CoCoA(1–190) in binding to the CoCoA C-terminal AD (Fig. 5A), inhibiting transactivation by the CoCoA C-terminal AD (Fig. 5B), and inhibiting the stimulation of CoCoA C-terminal AD by p300 (Fig. 5C) are consistent with our conclusions that the N-C interaction inhibits the activity of CoCoA C-terminal AD and that sumoylation at K29 enhances the activity of the C-terminal AD by preventing the N-C interaction and thus allowing p300 to bind to the C-terminal AD and enhance its activity.

The above results suggest that sumoylation of CoCoA should decrease its coactivator function for LEF1/β-catenin (where the N-terminal AD is required) but should increase its coactivator activity for nuclear receptors (where the C-terminal AD is required). We therefore examined the effects of sumoylation on the coactivator activity of full length CoCoA for LEF1 and estrogen receptor (ER). Wild type CoCoA dramatically enhanced LEF1 reporter activity (Fig. 5D) and ER reporter activity (Fig. 5E) in the presence of β-catenin and GRIP1, respectively. Consistent with our previous experiments (Fig. 4), fusion of SUMO1 to CoCoA caused an increase in the level of CoCoA protein expressed, by about 2-fold in this case, presumably by stabilizing the protein (Fig. 5D &5E, bottom panels). As expected, the coactivator activity of CoCoA for LEF1 was decreased when CoCoA was fused with SUMO1 (Fig. 5D), especially when assays containing similar amounts of CoCoA and SUMO1-CoCoA are compared (e.g. assays 5 & 6). This is consistent with the inhibition of the N-terminal AD of CoCoA by fusion of SUMO1. In contrast, the SUMO1-CoCoA had coactivator activity greater than that of CoCoA, consistent with our finding that SUMO fusion to the N-terminus of CoCoA prevents it from binding to and inhibiting the C-terminal AD. Taken together, these results suggest that sumoylation is a mechanism that differentially regulates the coactivator activity of CoCoA for LEF1 and ER.

## Discussion

### Sumoylation of CoCoA

Post-translational modification of proteins is a widely used mechanism for regulation of a variety of cellular processes. In the present study, we have shown that CoCoA is covalently modified by SUMO1 and SUMO2 in intact cells with PIAS1 and ARIP3 as the E3 ligases (Fig. 1, 2, 3). Lys-29 at the N-terminus of CoCoA is the major sumoylation site, because the K29R mutant was modified only poorly. Lys-52 may be a minor sumoylation site, since the small amount of sumoylation of the K29R mutant disappeared in the K29R, K52R double mutant (Fig. 3). The K29 sumoylation site is fully conserved from human to rat, and also present in a CoCoA homologue, the *Xenopus *nuclear dot protein NDP52. Lys-52 is conserved among CoCoA and its homologues NDP52 and TAX1BP1, but the consensus sumoylation motif surrounding K52 is only conserved in CoCoA from human to rat.

The small relative proportion of sumoylated CoCoA that we observed, even in the presence of an over-expressed E3 ligase (Fig. 2), suggests that only a very small fraction of total CoCoA is sumoylated in the cell at any given time. Whether the amount of CoCoA which is sumoylated can be regulated by external signals, and whether the sumoylation of CoCoA occurs only in certain sub-cellular locations are questions which remain to be investigated.

Some lysine residues in proteins can be modified either by sumoylation or by another type of post-translational modification, such as acetylation, methylation, or ubiquitylation. In such cases, sumoylation would prevent other post-translational modifications of the same lysine. In the case of IκBα [25] and Mdm2 [26], sumoylation appears to block ubiquitylation of these proteins and prevents proteasome-dependent degradation. Acetylation of the oncogene pleomorphic adenoma gene 1 (PLAG1) by p300 enhanced transactivation, while sumoylation on the same lysine residue appears to have a repressive effect on transactivation due to regulation of subnuclear localization [27]. Other post-translational modifications have also been shown to be involved in regulation of protein sumoylation. Phosphorylation of MEF2C, a member of the myocyte enhance factor 2 family, has been shown to enhance SUMO-conjugation at a site in close proximity to the phosphorylation site [28]. The human CoCoA was recently found to be an *in vitro *substrate of p42 MAPK and of CDK2/Cyclin E with predicted phosphorylation sites in both the N- and C-terminal activation domains [29]. However, we did not find any known phosphorylation-dependent sumoylation motifs near the major K29 sumoylation site or the possible minor K52 sumoylation site in CoCoA. Further studies on the various post-translational modifications of CoCoA, their functional interactions, and their biological roles are needed to delineate the complex regulation of CoCoA.

### Sumoylation regulates CoCoA AD activity

Sumoylation is involved in the regulation of many cellular processes. SUMO-modification of transcription factors is frequently connected to transcriptional repression. In some cases the transcriptional repression is due to sumoylation-induced alteration in cellular localization of the target proteins. For example, PIASy stimulation of LEF1 sumoylation in the Wnt signaling pathway results in sequestering of LEF1 to the PML nuclear bodies, which causes inhibition of LEF1-transcription [12]. Sumoylation has also been shown to regulate protein-protein interactions, resulting in transcriptional repression. For instance, sumoylation of p300 and the ETS domain transcription factor Elk-1 leads to transcriptional repression through recruitment of HDAC6 [30] and HDAC2 [31], respectively. SUMO-modification of several transcription factors, including androgen receptor [32], glucocorticoid receptor [33], ETS-1 [34], Pax3 [35], and Smad4 [36], has also been shown to recruit the transcriptional corepressor Daxx. However, exceptions have also been found to the generally negative effect of sumoylation in transcriptional regulation. Sumoylation of GRIP1 has been shown to promote its interaction with nuclear receptors and enhance its coactivator function. Mutation of the SUMO-modification site attenuated the ability of GRIP1 to enhance androgen receptor-dependent transcription [24].

CoCoA contains two known transcriptional ADs. The N-terminal AD of CoCoA is required for the coactivator function of CoCoA in the LEF1/β-catenin-mediated Wnt signaling pathway. The C-terminal AD of CoCoA is required for the coactivator function of CoCoA with nuclear receptors. The utilization of different ADs by CoCoA in different coactivator complexes may be regulated through post translational modifications. Here we fused SUMO1 to the N-terminal end of CoCoA to mimic the natural sumoylation event at K29. While the N-terminal fusion does not necessarily replicate the exact phenotype of natural sumoylation, it has been used as a model for the functional effects of sumoylation. Our results with this SUMO fusion model suggest that sumoylation of CoCoA regulates the differential use of the ADs. Mimicking sumoylation by fusing SUMO1 to CoCoA or CoCoA(1–190) attenuated the N-terminal AD activity and the coactivator activity in β-catenin/LEF1-mediated transcriptional activation (Fig. 4 &5D). This reduced secondary coactivator activity may be due to the sumoylation-induced reduction in the interaction of the N-terminal AD with p300 (Fig. 4B), which contributes to the N-terminal AD activity in the β-catenin/LEF1 coactivator complex [20].

On the other hand, the regulation of the C-terminal AD by sumoylation appears to be more complex. The C-terminal AD is required for CoCoA coactivator function with nuclear receptors. When CoCoA functions as a coactivator for nuclear receptors, p300 has been shown to interact with and contribute to transcriptional activation by the C-terminal AD of CoCoA [22]. Since sumoylation of CoCoA N-terminus reduces the interaction of CoCoA N-terminus with p300 (Fig. 4B), this may increase the availability of p300 for contributing to the activity of the CoCoA C-terminal AD. In addition, we demonstrated an interaction between N- and C-termini of CoCoA (Fig. 5A) and showed that this interaction attenuated the activity of the C-terminal AD (Fig. 5B), apparently by preventing interaction of the C-terminal AD with p300 (Fig. 5C). Fusion of SUMO1 to the CoCoA N-terminal fragment (mimicking sumoylation) disrupted the N-C interaction (Fig. 5A) and prevented the negative regulation of the C-terminal AD by the N-terminus (Fig. 5B–C). Furthermore, as predicted from the above results, fusion of SUMO1 to full length CoCoA enhanced its coactivator activity with ER and GRIP1, where the CoCoA C-terminal AD is required (Fig. 5E). While regulation of protein-protein interaction and transcriptional repression by sumoylation have been widely demonstrated, the use of sumoylation for differential utilization of transcriptional ADs in a single protein is a novel concept.

How can sumoylation of such a small fraction of total cellular CoCoA have any effect on the activity of CoCoA? One possibility is that certain external signals might result in a large increase in the fraction of total cellular CoCoA that is sumoylated. We would expect an increase in sumoylation of CoCoA to favor nuclear receptor function at the expense of LEF1/β-catenin-mediated transcription, and this was borne out by our results (Fig. 5D &5E). Another possibility is that CoCoA is only sumoylated under specific circumstances where the activity of the C-terminal AD is required. The fact that over-expression of E3 ligases enhances the sumoylation of CoCoA (Fig. 2) suggests that the E3 ligases are limiting and that sumoylation of CoCoA may be regulated by controlling access of the E3 ligases to CoCoA. Some of the SUMO E3 ligases have been shown to function as coactivators for nuclear receptors [24]. Therefore, we propose that sumoylation of CoCoA may occur when CoCoA and PIAS1 or ARIP3 are recruited by hormone-activated nuclear receptors to a specific target gene promoter. The juxtaposition of CoCoA with an E3 ligase in the coactivator complex could result in an efficient localized sumoylation of CoCoA at K29. This sumoylation would prevent intra-molecular binding between the N- and C-termini of CoCoA and thereby relieve repression of the C-terminal AD of CoCoA by the N-terminal AD. Perhaps only a transient disruption of the N-C interaction is required to allow the C-terminal AD of CoCoA to carry out its coactivator function.

## Conclusion

The studies shown here demonstrated the potential role of SUMO1 modification in regulating the transcriptional activity of CoCoA. The transcriptional coactivator CoCoA utilizes two different ADs to function in various regulatory contexts on different promoters. The binding of CoCoA to p160 coactivators (in nuclear receptor-mediated gene regulation) versus β-catenin (in LEF1-mediated gene regulation) appears to dictate which of the CoCoA ADs is used for transcriptional activation. Our results suggest that sumoylation of CoCoA differentially regulates the activities of the ADs by attenuating the N-terminal AD activity, inhibiting the interaction between the CoCoA N- and C-termini, and thus enhancing the activity of the C-terminal AD. Our results also suggest that CoCoA sumoylation therefore enhances the coactivator activity of CoCoA with nuclear receptors while it inhibits the coactivator function of CoCoA with β-catenin and LEF1.

## Methods

### Plasmids

pSG5.HA-CoCoA, pSG5.HA-CoCoA(1–190), pSG5.HA-CoCoA(1–500), pSG5.HA-CoCoA(150–500), pSG5.HA-CoCoA(470–691), pGEX-5X1.CoCoA(470–691), pM.CoCoA(1–149), pM.CoCoA(1–190), pSG5.HA-GRIP1, pHE0, and MMTV(ERE)-LUC were described previously [16,22]. pM.CoCoA(1–190, K29R), pSG5.HA-CoCoA(K29R), pSG5.HA-CoCoA(K52R) and pSG5.HA-CoCoA(K29R, K52R) were made using the Quick Change Site-Directed Mutagenesis kit (Stratagene) following the manufacturer's instructions. Plasmids expressing EGFP-SUMO1, Myc-SUMO2, PIAS1 and ARIP3 were kind gifts from Dr. David Ann (City of Hope, Duarte, California) and Jorma Palvimo (University of Helsinki, Finland). SUMO1 fusion protein was constructed by inserting a PCR amplified CoCoA full length, (1–190) or (1–190, K29R) into the XhoI/BglII site of a pSG5HA.SUMO1 mutant which lacks the two tandem glycines at the C-terminus of processed SUMO1.

### Cell culture and transfections

CV-1, 293T and COS-7 cells were maintained in Dulbecco's modified Eagle's medium (DMEM) with 10% fetal bovine serum and penicillin and streptomycin. For reporter gene assays, CV-1 and 293T cells were transiently transfected in 12-well or 24-well plates and assayed for luciferase activity as described previously [16]. Mean and standard deviation of triplicate points are shown for one representative transfection experiment which is representative of multiple independent experiments.

### Protein-protein interaction assay

GST pull-down assays were performed as described previously [16] using either in vitro translated protein or cell extracts from COS-7 cells transiently transfected with the indicated plasmids. The bound proteins were eluted from the beads and analyzed by immunoblot with anti-HA antibody (Roche Applied Science).

### In vivo and in vitro sumoylation assay

293T or COS-7 cells were transfected with the indicated plasmids encoding proteins with an HA-tag. Cell extracts were collected in the presence of 20 mM N-ethylmaleimide 40 h post-transfection. Immunoblots were performed with anti-HA antibody. The in vitro sumoylation assay was performed with a SUMOylation kit (BIOMOL International, LP) using in vitro translated ^35^S-labeled protein as substrates.

## Abbreviations

AD, activation domain; ARIP, androgen receptor interacting protein; ARNT, aryl hydrocarbon receptor nuclear translocator; CARM1, coactivator associated arginine methyltransferase 1; CBP, CREB binding protein; CDK, cyclin dependent kinase; CoCoA, coiled-coil coactivator; DBD, DNA binding domain; EGFP, enhanced green fluorescent protein; GRIP1, glucocorticoid receptor interacting protein 1; GST, glutathione S-transferase; HA, hemagglutinin epitope; HDAC, histone deacetylase; IκBα, inhibitor of NFκB; LEF, lymphocyte enhancer factor; MAPK, mitogen-activated protein kinase; Mdm2, transformed double minute 2; MEF2C, myocyte enhancer factor 2C; NDP52, nuclear dot protein 52; PIAS, protein inhibitor of activated STAT; PLAG1, pleomorphic adenoma gene 1; PML, promyelocytic leukemia protein; STAT, signal transducers and activators of transcription; SUMO, small ubiquitin-like modifier; TAX1BP1, TAX1 binding protein; TCF, T cell factor.

## Authors' contributions

CKY, JHK and MRS conceived and designed the experiments; CKY and JHK performed the experiments and analyzed the data; DKA supplied technical expertise; CKY, JHK and MRS drafted the manuscript; all authors have approved the manuscript.

## References

[B1] Muller S, Hoege C, Pyrowolakis G, Jentsch S (2001). Sumo, ubiquitin's mysterious cousin. Nature Reviews Molecular Cell Biology.

[B2] Poukka H, Aarnisalo P, Karvonen U, Palvimo JJ, Janne OA (1999). Ubc9 interacts with the androgen receptor and activates receptor-dependent transcription. J Biol Chem.

[B3] Seeler JS, Dejean A (2003). Nuclear and unclear functions of SUMO. Nature Reviews Molecular Cell Biology.

[B4] Kamitani T, Kito K, Nguyen HP, Fukuda-Kamitani T, Yeh ETH (1998). Characterization of a second member of the sentrin family of ubiquitin-like proteins. J Biol Chem.

[B5] Kagey MH, Melhuish TA, Wotton D (2003). The polycomb protein Pc2 is a SUMO E3. Cell.

[B6] Kirsh O, Seeler JS, Pichler A, Gast A, Muller S, Miska E, Mathieu M, Harel-Bellan A, Kouzarides T, Melchior F, Dejean A (2002). The SUMO E3 ligase RanBP2 promotes modification of the HDAC4 deacetylase. EMBO J.

[B7] Johnson ES (2004). Protein modification by SUMO. Annual Review of Biochemistry.

[B8] Schmidt D, Muller S (2003). PIAS/SUMO: new partners in transcriptional regulation. Cell Mol Life Sci.

[B9] Megidish T, Xu JH, Xu CW (2002). Activation of p53 by protein inhibitor of activated Stat1 (PIAS1). J Biol Chem.

[B10] Rogers RS, Horvath CM, Matunis MJ (2003). SUMO modification of STAT1 and its role in PIAS-mediated inhibition of gene activation. J Biol Chem.

[B11] Ungureanu D, Vanhatupa S, Kotaja N, Yang J, Aittomaki S, Janne OA, Palvimo JJ, Silvennoinen O (2003). PIAS proteins promote SUMO-1 conjugation to STAT1. Blood.

[B12] Sachdev S, Bruhn L, Sieber H, Pichler A, Melchior F, Grosschedl R (2001). PIASy, a nuclear matrix-associated SUMO E3 ligase, represses LEF1 activity by sequestration into nuclear bodies. Genes & Development.

[B13] Poukka H, Karvonen U, Janne OA, Palvimo JJ (2000). Covalent modification of the androgen receptor by small ubiquitin-like modifier 1 (SUMO-1). Proc Natl Acad Sci U S A.

[B14] Sentis S, Le Romancer M, Bianchin C, Rostan MC, Corbo L (2005). Sumoylation of the estrogen receptor alpha hinge region regulates its transcriptional activity. Mol Endocrinol.

[B15] Tian S, Poukka H, Palvimo JJ, Janne OA (2002). Small ubiquitin-related modifier-1 (SUMO-1) modification of the glucocorticoid receptor. Biochem J.

[B16] Kim JH, Li H, Stallcup MR (2003). CoCoA, a nuclear receptor coactivator which acts through an N-terminal activation domain of p160 coactivators. Mol Cell.

[B17] Kim JH, Stallcup MR (2004). Role of the Coiled-coil Coactivator (CoCoA) in Aryl Hydrocarbon Receptor-mediated Transcription. J Biol Chem.

[B18] Yang CK, Kim JH, Li H, Stallcup MR (2006). Differential use of functional domains by coiled-coil coactivator in its synergistic coactivator function with beta-catenin or GRIP1. J Biol Chem.

[B19] Morin PJ (1999). beta-catenin signaling and cancer. Bioessays.

[B20] Yang CK, Kim JH, Stallcup MR (2006). Role of the N-terminal activation domain of the coiled-coil coactivator in mediating transcriptional activation by beta-catenin. Mol Endocrinol.

[B21] Stallcup MR, Kim JH, Teyssier C, Lee YH, Ma H, Chen D (2003). The roles of protein-protein interactions and protein methylation in transcriptional activation by nuclear receptors and their coactivators. J Steroid Biochem Mol Biol.

[B22] Kim JH, Yang CK, Stallcup MR (2006). Downstream signaling mechanism of the C-terminal activation domain of transcriptional coactivator CoCoA. Nucleic Acids Research.

[B23] Tojo M, Matsuzaki K, Minami T, Honda Y, Yasuda H, Chiba T, Saya H, Fujii-Kuriyama Y, Nakao M (2002). The aryl hydrocarbon receptor nuclear transporter is modulated by the SUMO-1 conjugation system. J Biol Chem.

[B24] Kotaja N, Vihinen M, Palvimo JJ, Janne OA (2002). Androgen receptor-interacting protein 3 and other PIAS proteins cooperate with glucocorticoid receptor-interacting protein 1 in steroid receptor-dependent signaling. J Biol Chem.

[B25] Desterro JMP, Rodriguez MS, Hay RT (1998). SUMO-1 modification of I kappa B alpha inhibits NF-kappa B activation. Molecular Cell.

[B26] Buschmann T, Fuchs SY, Lee CG, Pan ZQ, Ronai Z (2000). SUMO-1 modification of Mdm2 prevents its self-ubiquitination and increases Mdm2 ability to ubiquitinate p53. Cell.

[B27] Zheng G, Yang YC (2005). Sumoylation and acetylation play opposite roles in the transactivation of PLAG1 and PLAGL2. J Biol Chem.

[B28] Kang J, Gocke CB, Yu H (2006). Phosphorylation-facilitated sumoylation of MEF2C negatively regulates its transcriptional activity. BMC Biochem.

[B29] Wiemann S, Arlt D, Huber W, Wellenreuther R, Schleeger S, Mehrle A, Bechtel S, Sauermann M, Korf U, Pepperkok R, Sultmann H, Poustka A (2004). From ORFeome to biology: a functional genomics pipeline. Genome Res.

[B30] Girdwood D, Bumpass D, Vaughan OA, Thain A, Anderson LA, Snowden AW, Garcia-Wilson E, Perkins ND, Hay RT (2003). p300 transcriptional repression is mediated by SUMO modification. Molecular Cell.

[B31] Yang SH, Sharrocks AD (2004). SUMO promotes HDAC-mediated transcriptional repression. Molecular Cell.

[B32] Lin DY, Fang HI, Ma AH, Huang YS, Pu YS, Jenster G, Kung HJ, Shih HM (2004). Negative modulation of androgen receptor transcriptional activity by Daxx. Mol Cell Biol.

[B33] Lin DY, Lai MZ, Ann DK, Shih HM (2003). Promyelocytic leukemia protein (PML) functions as a glucocorticoid receptor Co-activator by sequestering Daxx to the PML oncogenic domains (PODs) to enhance its transactivation potential. J Biol Chem.

[B34] Li RZ, Pei HP, Watson DK, Papas TS (2000). EAP1/Daxx interacts with ETS1 and represses transcriptional activation of ETS1 target genes. Oncogene.

[B35] Hollenbach AD, Sublett JE, McPherson CJ, Grosveld G (1999). The Pax3-FKHR oncoprotein is unresponsive to the Pax3-associated repressor hDaxx. EMBO J.

[B36] Chang CC, Lin DY, Fang HI, Chen RH, Shih HM (2005). Daxx mediates the small ubiquitin-like modifier-dependent transcriptional repression of Smad. J Biol Chem.

